# Ciphertext-only attack on optical cryptosystem with spatially incoherent illumination: from the view of imaging through scattering medium

**DOI:** 10.1038/srep41789

**Published:** 2017-01-31

**Authors:** Meihua Liao, Wenqi He, Dajiang Lu, Xiang Peng

**Affiliations:** 1 Key Laboratory of Optoelectronic Devices and Systems of Ministry of Education and Guangdong Province, College of Optoelectronics Engineering, Shenzhen University, Shenzhen 518060, China

## Abstract

Security analysis is important and necessary for a new cryptosystem. In this paper, we evaluate the security risk of the optical cryptosystem with spatially incoherent illumination from the view of imaging through scattering medium and then demonstrate that it is vulnerable to ciphertext-only attack. The proposed ciphertext-only attack method relies on the optical memory effect for speckle correlations, which reveals a fact that the ciphertext’s autocorrelation is essentially identical to the plaintext’s own autocorrelation. Furthermore, by employing of an improved dynamic hybrid input-output phase-retrieval algorithm, we show that a plaintext image can be directly reconstructed from the autocorrelation of its corresponding ciphertext without any prior knowledge about the plaintext or the phase keys. Meanwhile, the theory analysis and experiment results will also be provided to verify the validity and feasibility of our proposed ciphertext-only attack method. To the best of our knowledge, this is the first time to report optical cryptanalysis from the point of view of imaging through scattering medium and we believe this contribution will open up an avenue to deepen the investigation of optical cryptosystems.

Since double random phase encoding (DRPE) was proposed by Refregier and Javidi^1^, optical cryptography has drawn a lot of attentions in the past two decades^2^,^3^,^4^,^5^,^6^,^7^,^8^,^9^,^10^,^11^,^12^,^13^,^14^,^15^,^16^,^17^ due to the fact that optical techniques have inherent advantages such as parallel signal processing and high dimensional operation. In the classical DRPE scheme, two statistically independent random phase masks are used as the security keys to scramble original plaintext data into a stationary white noise. Henceforth, numerous versions of DRPE scheme have been developed in different transform domains such as fractional Fourier domain^4^,^5^, Fresnel domain^6^, gyrator domain^7^ and fractional Mellin transform^8^. Meanwhile, researchers also invented a series of alternative optical cryptosystems, by taking advantage of various typical optical principles or architectures such as joint transform correlator architecture^9^, interference^10^,^11^, diffractive imaging^12^,^13^, computational ghost imaging^14^,^15^, ptychography^16^ and compressive sensing^17^. Nevertheless, these aforementioned cryptosystems are mostly working under coherent illumination and thus suffering from high sensitivity to misalignment and coherent artifact noise. Recently, Zang *et al*. have presented a simple and efficient spatially incoherent optical cryptosystem with only one imaging lens and one random phase mask (RPM) and claimed that it could efficiently decrease the errors generated from misalignment and coherent artifact noise^18^. Besides, the output ciphertext of this cryptosystem is an intensity pattern which can be directly and easily stored and transmitted through a common communication link.

As all we know, the security performance of a cryptosystem is of the major concern. A cryptosystem can be claimed to be secure enough only if it can endure the safety evaluation by cryptanalysis. Cryptanalysis refers to the study of cryptosystems with a view to finding any weaknesses in them that will permit retrieval of the plaintext from the ciphertext, without necessarily knowing the secret key. Cryptography and cryptanalysis are mutual support and mutual promotion to each other. With the rapid development of optical cryptography optical cryptanalysis is also attracting more and more attentions^19^,^20^,^21^,^22^,^23^,^24^,^25^,^26^. Meanwhile, various attacks on existing optical cryptosystems can further promote the invention of security-enhanced optical cryptosystems^27^,^28^,^29^,^30^,^31^.

In this manuscript we try to dig out the security leaks of Zang’s scheme for cryptanalysis. First of all, we would like to analyze its encryption mechanism. In essence, Zang’s method, like most of current optical encryption methods, largely depends on the RPM which is utilized to introduce the concept of “confusion” and “diffusion”. When a parallel beam goes through a RPM the output light will no longer propagates along their original direction, which means a scattering occurs. As shown in Fig. 1, the resulting scattering angle *θ* is determined by the phase mask gradient ΔΦ of the RPM^32^. That is to say, when a wavefront carrying the information of input image (plaintext) passes through a RPM (secret key), it could efficiently be disturbed due to the scattering effect caused by the RPM and form a speckle pattern (ciphertext) in the recording plane.

According to Kerckhoffs’ principle, the way to reveal the security flaw of Zang’s cryptosystem would be trying to directly recover the input image (plaintext) from the corresponding speckle pattern (ciphertext) without knowing the distribution of RPM (secret key). This way is called ciphertext-only attack (COA), which means a minimum resource is needed to break out the cryptosystem. It is worthwhile to note that this particular COA issue happens to be equivalent to a problem of imaging through scattering medium^33^. It is common sense that a detector could not get a clear image of the target object which is placed behind a scattering medium. Many researchers have made continuous efforts to study this problem and developed a series of methods for seeing through turbid media^32^,^33^,^34^,^35^,^36^,^37^,^38^. One of typical work was presented by Bertolotti *et al*., who successfully observed a fluorescent object hidden behind an opaque scattering layer by scanning the angle of illumination light^36^. Soon afterwards, Katz *et al*. improved this method and restored a real-valued object hidden behind a thin scattering layer by just one speckle pattern^37^.

Inspired by recent advances in imaging through scattering medium^33^,^36^,^37^,^38^, we proposed a COA against the optical cryptosystem with spatially incoherent illumination (i.e. Zang’s scheme). The proposed COA method relies on the optical memory effect for speckle correlations, which reveals a fact that the ciphertext’s autocorrelation is essentially identical to the plaintext’s own autocorrelation. Furthermore, by employing an improved dynamic hybrid input-output phase-retrieval algorithm, we can then reconstruct the plaintext image from the autocorrelation of its corresponding ciphertext without knowing about the phase keys.

## Principle

### Overview of the optical cryptosystem with spatially incoherent illumination

**Figure 2:**
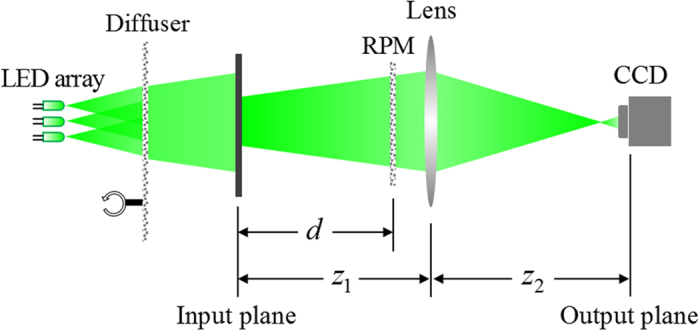
Scheme setup of optical cryptosystem with spatially incoherent illumination.

In this subsection, we briefly review the principle of the optical cryptosystem with spatially incoherent illumination^18^. The encryption process is implemented by an optical configuration. As shown in Fig. 2, the plaintext is placed at the input plane. A RPM and an imaging lens are placed at the distances *d* and *z*_1_ from the input plane, respectively. When the whole system is illuminated by spatially incoherent light, an intensity pattern (ciphertext) is captured by a CCD camera at the output plane. The distance between the output plane and the imaging lens is *z*_2_. This encryption system can be regarded as an incoherent imaging system and the encryption process could be expressed as


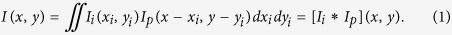


where *I*_*i*_(*x*_*i*_, *y*_*i*_) and 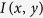 denote the plaintext and ciphertext, respectively, *I*_*p*_(*x, y*) is the incoherent intensity point spread function (PSF) of the system, and the symbol “*” stands for the convolution operation.

The decryption process could be implemented by optical or digital means. The detailed steps for decryption are as follows: First, replacing the original plaintext with a point source (i.e. Dirac function) located on the center of the input plane. A diverging spherical wave is generated when illuminating the input plane and then it travels through the same path to form an intensity pattern *I*_*p*_(*x, y*), which is recorded on the output plane by an intensity camera (such as charge coupled device (CCD)) as the PSF of this cryptosystem. Second, storing this PSF *I*_*p*_(*x, y*) and transmitting it to the authorized receiver. Third, calculating out the plaintext *I*_*i*_(*x*_*i*_, *y*_*i*_) from the ciphertext 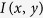 with the received PSF *I*_*p*_(*x, y*) by a simple deconvolution operation as


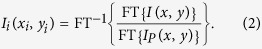


where FT{⋅} and FT^−1^{⋅} represent the Fourier transform operation and the inverse Fourier transform operation, respectively.

### Security analysis and ciphertext-only attack process

Before preforming the COA, we analyze the security risk of this optical cryptosystem with spatially incoherent illumination from the point of view of imaging through scattering medium. According to the eq. (1), the ciphertext 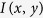 is given by a convolution of the plaintext *I*_*i*_(*x*_*i*_, *y*_*i*_) with the incoherent PSF *I*_*p*_(*x, y*) of the imaging system. Taking the autocorrelation of 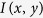 and making a further deduction by using the convolution theorem, we have


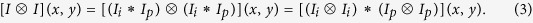


where the symbol “⊗” denotes the autocorrelation operation. As the autocorrelation of the PSF, [*I*_*p*_⊗*I*_*p*_](*x, y*), is a sharply peaked function^24^,^25^ (essentially the autocorrelation of broadband noise), the right hand side of eq. (3) is effectively equal to [*I*_*i*_⊗*I*_*i*_](*x, y*), then eq. (3) can be approximated by following equation


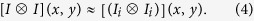


Theoretically, the reason why the autocorrelation of the ciphertext is essentially identical to the autocorrelation of plaintext is the intrinsic isoplanatism that arises from the optical memory effect for speckle correlation^38^. The optical memory effect states that the light from nearby points on the plaintext is scattered by the RPM will be a pair of highly correlated but shifted speckle patterns formed on the output plane^33^. For spatially incoherent illumination, the ciphertext is simply a superposition of these identical shifted speckle patterns. That means that the autocorrelation property is also transmitted from the plaintext to ciphertext with high fidelity. Therefore the autocorrelation of plaintext could be directly obtained from only the ciphertext by eq. (4). Although we could not directly recover the plaintext itself from its autocorrelation we are indeed able to recover it with the help of an iterative phase-retrieval algorithm^39^.

Here, we show details of this COA approach, which can retrieve the plaintext with only ciphertext. Given an arbitrarily intercepted ciphertext 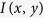, what we need is to calculate its autocorrelation by taking an inverse Fourier transform of its power spectrum according to the Wiener–Khinchin theorem. It can be mathematically expressed as


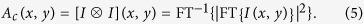


where 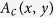 denotes the autocorrelation of 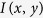. It also approximately equals to the autocorrelation of the plaintext according to eq. (4). Thus, we can get the corresponding power spectrum 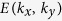 by performing a Fourier transform on both sides of eq. (5)


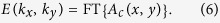


where *k*_*x*_ and *k*_*y*_ are the coordinates in spatial frequency domain. Thus, the issue to be solved in proposed COA approach can be converted to a phase-retrieval problem with single intensity. As we known, the hybrid input-output (HIO) phase-retrieval algorithm not only requires the amplitude constraint in the transform domain but also the support constraint in the object domain. Therefore, we must estimate the support constraint in the object domain from the calculated power spectrum of the plaintext. In our approach the number of nonzero pixel (NNP) constraint has been introduced as a dynamic support constraint in the object domain^40^,^41^, and then an effective dynamic HIO (DHIO) algorithm is developed to solve the problem of phase-retrieval with single intensity distribution. By employing this improved DHIO phase-retrieval algorithm, we can directly reconstruct the plaintext image from the autocorrelation of its corresponding ciphertext without any prior knowledge about the plaintext or the phase keys.

## Results

**Figure 3:**
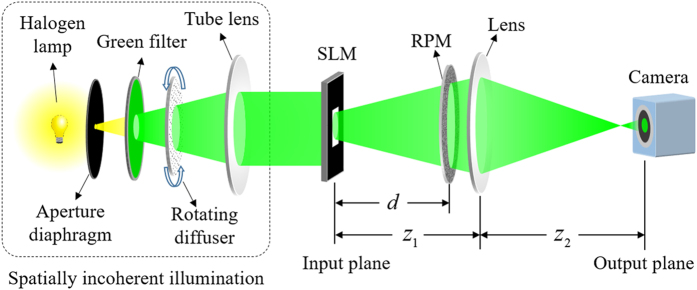
Schematic of the optical experimental set-up for the verification of proposed ciphertext-only attack approach.

The optical experiments were carried out to verify the proposed COA approach discussed in last subsection. The experimental set-up is schematically shown in Fig. 3. In our experiments the plaintext image to be encrypted was placed at distance of *d* behind the RPM and was illuminated by a narrowband spatially incoherent pseudothermal source (composed of a halogen lamp, an aperture diaphragm, a green filter, a rotating diffuser and a tube lens). The ciphertext images was recorded by a high-resolution CMOS camera.

**Figure 4:**
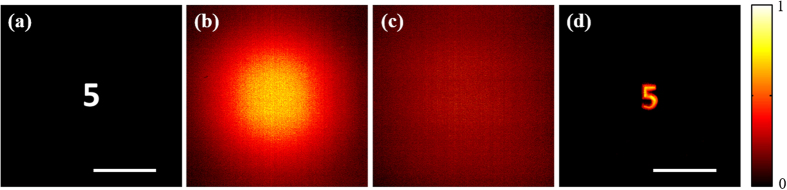
The experimental results of optical cryptosystem with spatially incoherent illumination. (**a**) The plaintext. (**b**) The ciphertext. (**c**) The incoherent intensity point spread function. (**d**) The decrypted result. Scale bars: Scale bars: 200 camera pixels, corresponding to 1.1 mm.

Firstly, we verified the effectiveness of experimental set-up by performing the encryption and decryption processes of optical cryptosystem with spatially incoherent illumination. A binary image with the numeric character “5”, as shown in Fig. 4(a), was loaded on the SLM as the plaintext of cryptosystem. The corresponding ciphertext is shown in Fig. 4(b). Meanwhile a pinhole with 20 μm in diameter served a point source. In order to obtain the PSF of imaging system, the pinhole image was loaded on the SLM again. The obtained PSF is shown in Fig. 4(c), and the decrypted result is shown in Fig. 4(d).

**Figure 5:**
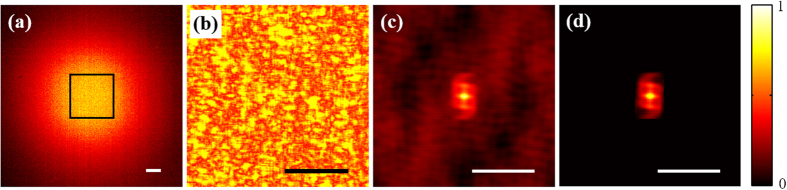
The experimental results of the proposed ciphertext-only attack. (**a**) The given ciphertext (same to Fig. 4(b)). (**b**) The central rectangular area of (**a**). (**c**) The autocorrelation of (**b**). (**d**) The processed autocorrelation. Scale bars: 200 camera pixels, corresponding to 1.1 mm.

Then, we implemented the proposed COA with the data obtained from this experimental set-up. Suppose we are given a ciphertext shown in Fig. 5(a), actually it is exactly the same with Fig. 4(b). We first extracted its central rectangular area (Fig. 5(b)) to adapt the real size of the plaintext. The autocorrelation of Fig. 5(b) was then calculated by eq. (5) as shown in Fig. 5(c). Then Fig. 5(c) was cropped to a rectangular window and the minimum pixel brightness in this window was background-subtracted from the entire autocorrelation trace. In addition the intensity value of central pixel of the autocorrelation was taken as equal to one of its neighbors. After image processing we obtain the correlation as shown in Fig. 5(d).

Next, we sought all pixels whose values were smaller than a pre-determined threshold value (e.g. 0.0002) in Fig. 5(d), and set all those values to be zero. This processed image could also be regarded as practical autocorrelation pattern of the plaintext and it was directly used in the DHIO algorithm. By doing this, we can easily get the NNP of the autocorrelation pattern of plaintext. Meanwhile, according to a thumb of rule that the object’s NNP is usually between 1/6 and 1/4 of its autocorrelation’s NNP^42^,^43^, we can then get an estimation range of plaintext’s NNP, in which a few NNP values are selected to help determining the object domain constrains in the aforementioned DHIO algorithm, respectively. And then we choose the most highly recognizable images from all the results as the final retrieved image.

**Figure 6:**
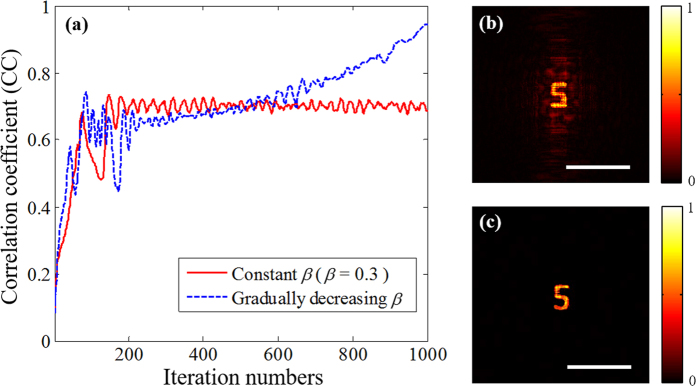
Performances of the proposed two types of DHIO phase-retrieval algorithm. (**a**) Convergence curve (red solid line represent the condition with constant *β* and blue dashed line represent the condition with gradually decreasing *β*). (**b**) The retrieved result with constant *β* (the first type). (**c**) The retrieved result with gradually decreasing *β* (the second type). Scale bars: 200 camera pixels, corresponding to 1.1 mm.

At last, the plaintext image can be reconstructed from this obtained autocorrelation distribution. Here, we develop a dynamic hybrid input-output (DHIO) phase-retrieval algorithm (see Method section). As we known, the feedback parameter *β* in DHIO algorithm is very important because it controls the convergence properties of DHIO algorithm. Therefore, we also discuss the influence of the feedback parameter *β* by modifying aforementioned DHIO algorithm in two different points as shown in Fig. 6. First, the value of *β* is set to be a constant (*β* = 0.3). Second, the value of *β* is gradually decreasing from 1 to 0 in steps of 0.02. For each *β* value, 20 iterations of the algorithm was performed. The convergence of the two types of DHIO algorithm was monitored by calculating the correlation coefficient (CC) between |*G*_*k*_(*k*_*x*_, *k*_*y*_)| and 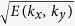
^2^. The CC is defined as follows:


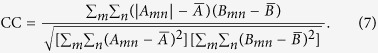


where 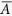 and 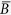 denote the mean value of images *A* and *B. A*_*mn*_ and *B*_*mn*_ are the pixel values at the coordinate (*m, n*) of images *A* and *B*, respectively. Obviously, the CC value ranges from 0 to 1, and the higher CC value implies the more similar between two images. Figure 6(a) shows two corresponding convergence curves wherein the abscissa represents the number of iterations and the ordinate represents the CC value. The red solid line and blue dashed line respectively represent the condition with constant *β* (the first type) and gradually decreasing *β* (the second type). After 1000^th^ iterations, the retrieved results with constant *β* and gradually decreasing *β* are shown in Fig. 6(b,c), respectively. It is obvious that both two pictures resemble the plaintext image and the second type has better convergence characteristics.

**Figure 7:**
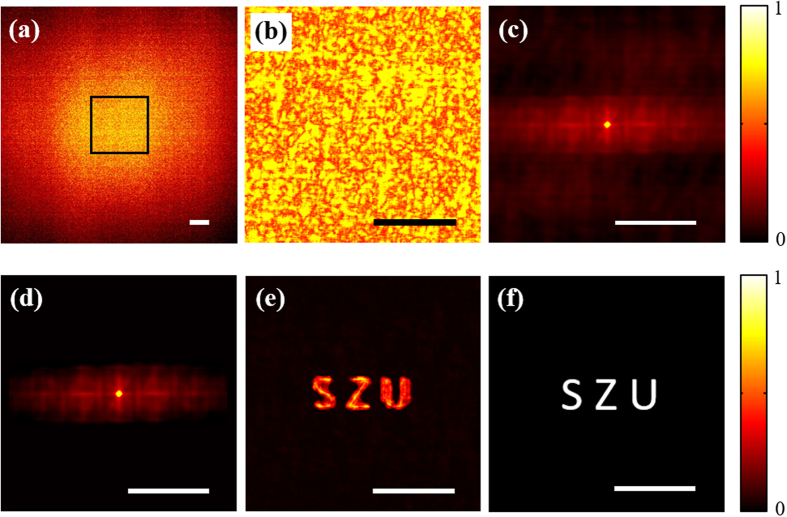
The reconstructed result of another ciphertext. (**a**) The ciphertext (the raw camera image). (**b**) The central part of (**a**). (**c**) The autocorrelation of (**b**). (**d**) The processed autocorrelation. (**e**) The retrieved result by COA. (**f**) The original plaintext including the letter “SZU”. Scale bars: 200 camera pixels, corresponding to 1.1 mm.

To further validate our approach, we recovered another plaintext including the letter “SZU” (letter height 400 um) from its ciphertext. The given ciphertext and its central part are shown in Fig. 7(a,b), respectively. They are low-contrast and seemingly random pattern with no visible relation to the true shape of the plaintext information. The autocorrelation pattern of Fig. 7(b) and the processed autocorrelation pattern are respectively shown in Fig. 7(c,d). The retrieved result by COA approach is shown in Fig. 7(e). As a reference the original plaintext image is shown in Fig. 7(f).

**Figure 8:**
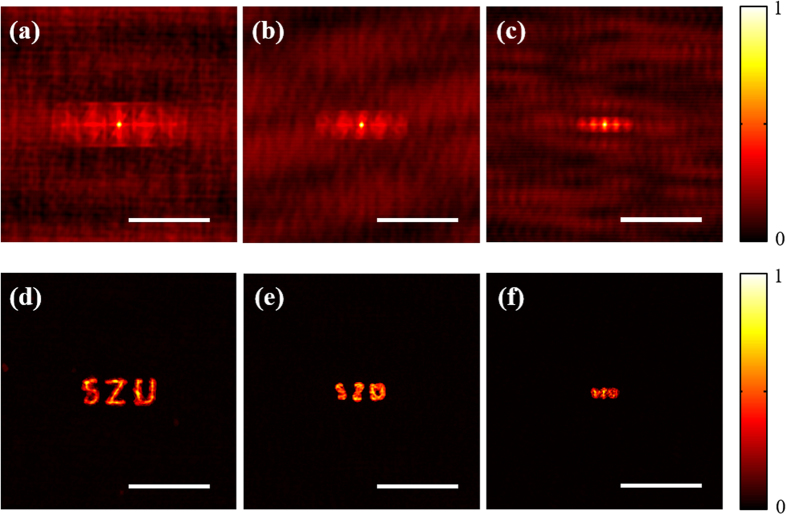
The reconstructed result of the smaller letters. (**a–c**) The measured autocorrelations for letter height 0.3 mm, 0.2 mm and 0.1 mm, respectively, (**d–f**) The corresponding restored image for letter height 0.3 mm, 0.2 mm and 0.1 mm, respectively. Scale bars: 200 camera pixels, corresponding to 1.1 mm.

Also, we noted that the letters were slightly distorted in the restored images (see Figs 6(c) and 7(e)). Furthermore, we’ve checked the validity of the proposed attack scheme for the same letter but in smaller sizes. We decreased the size of the letters “SZU” (see Fig. 7(f)) to its original 75%, 50% and 25% (the practical letter height are 0.3 mm, 0.2 mm and 0.1 mm, respectively). The corresponding measured autocorrelations and restored images are shown in Fig. 8(a–f), respectively. It is obvious that the distortion of the retrieved letters will get worse and worse as the size of the letters getting smaller and smaller. That means the attack would not be applicable if the size of letter height is less than 0.2 mm in the aforementioned experimental system.

The major reason for letter distortion is that the restored image is not the exact solution but an optimal one. The signal-to-noise ratio of the measured autocorrelation is vital to the proposed dynamic hybrid input-output phase-retrieval algorithm. However, the smaller size or denser distribution will definitely decrease the signal-to-noise ratio of the measured autocorrelation. The smaller size of the letter present in its plaintext image, the lower signal-to-noise ratio of the measured autocorrelation. This fact is clearly visible in the Fig. 8(a–c), where the information on the letter’s autocorrelation is contained in a small area on the center of a large background. Therefore, the corresponding retrieved image (letters) will be distorted and blurry with worse quality, as shown in Fig. 8(d–f).

## Conclusion and Discussion

We evaluate the security risk of the optical cryptosystem with spatially incoherent illumination from the point of view of imaging through scattering medium, We have demonstrate that it is vulnerable to proposed COA by taking advantage of the optical memory effect for speckle correlation. By performing proposed COA approach, an unauthorized user could directly retrieve the plaintext from an intercepted ciphertext.

It should be pointed out that there is some size limitations in the presented COA scheme. If the size of the original plaintexts is too small, the restored images will be distorted caused by the lower signal-to-noise ratio (SNR) of measured autocorrelation pattern. Meanwhile, the number of nonzero pixels (NNP) in plaintext image theoretically should be no greater than 25% of the total pixel numbers in the recorded ciphertext image because a two-dimension signal can be uniquely specified by the magnitude of its twice oversampled discrete Fourier transform^44^.

It is noteworthy that most existed optical cryptosystems are based on the scattering effect of one or several RPM(s). The PSFs of these cryptosystems are normally randomly distributed speckle patterns, which could result in their autocorrelation to be a sharply peaked function. This could then lead to a potential security flaw because one can retrieve the plaintext by making use of the relationship between the autocorrelation of plaintext and that of ciphertext. To the best of our knowledge, this is the first time to report optical cryptanalysis from the point of view of imaging through scattering medium and we believe this contribution will open up an avenue to deepen the investigation of optical cryptosystems.

## Methods

### Optical experimental set-up

The complete experimental set-up is presented in Fig. 3. A halogen source combined with a band pass filter (central wavelength *λ* = 550 nm) was introduced as a spatially incoherent illumination source. A rotating diffuser and a tube lens are placed before the input plane to ensure the beam to be totally incoherent and collimated. The focal length of the imaging lens is *f* = 150 mm and *z*_1_ = *z*_2_ = 2*f, d* ≈ 2*f*. The plaintext image is loaded on a SLM (Holoeye, LC2002) which was placed at the input plane and worked at the amplitude modulation mode. The RPM was a 220 grit-ground-glass diffuser (Thorlabs, DG10-220-MD). A high-resolution CMOS camera (Photonfocus, MV1-D2048-96-G2-10, resolution: 2048 × 2048 px, pixel size: 5.5 μm × 5.5 μm, active optical area: 11.26 mm × 11.26 mm) was placed at the output plane to capture the ciphertext (speckle pattern).

### Dynamic hybrid input-output phase-retrieval algorithm

**Figure 9:**
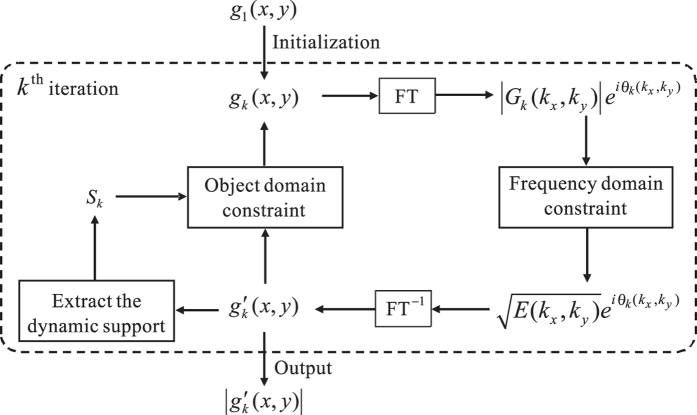
Block diagram for DHIO phase-retrieval algorithm with the number of nonzero pixel constraint in the *k*^th^ iteration.

A block-diagram of the proposed dynamic hybrid input-output phase-retrieval algorithm is illustrated in Fig. 9. Firstly, an arbitrarily generated image *g*_1_(*x, y*) is chosen as an initial input image (i.e. initial guess of the plaintext) in the object domain. Suppose the iteration algorithm proceeds to the *k*^th^ iteration, the following steps could be described as:

1. Perform the Fourier transform on the *k*^th^ input image *g*_*k*_(*x, y*) and obtain its frequency spectrum *G*_*k*_(*k*_*x*_, *k*_*y*_):
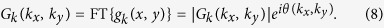
2. Impose the frequency magnitude constraint on *G*_*k*_(*k*_*x*_, *k*_*y*_), i.e., replace the modulus of *G*_*k*_(*k*_*x*_, *k*_*y*_) with 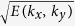:
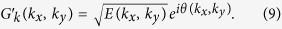
3. Perform the inverse Fourier transform *G*′_*k*_(*k*_*x*_, *k*_*y*_) and obtain a new complex amplitude distribution *g*′_*k*_(*x, y*) in the object domain:
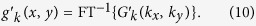
4. Take the modulus of *g*′_*k*_(*x, y*) and find out the positions holding the largest *N* pixel absolute values, which is regarded as current dynamic support *S*_*k*_, which varies in each iteration. Here, *N* is the estimated value of NPN, and we will introduce an estimation technique.
5. Impose the object domain constraint on *g*′_*k*_(*x, y*) to obtain a new input *g*_*k*+1_(*x, y*), which is:

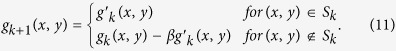


where *β* is a feedback parameter that controls the convergence properties of this algorithm. Repeat steps 1–5 until the pre-determined iteration times is reached, and the modulus pattern of the distribution in the object domain will be treated as the retrieved result.

## Additional Information

**How to cite this article**: Liao, M. *et al*. Ciphertext-only attack on optical cryptosystem with spatially incoherent illumination: from the view of imaging through scattering medium. *Sci. Rep.* **7**, 41789; doi: 10.1038/srep41789 (2017).

**Publisher's note:** Springer Nature remains neutral with regard to jurisdictional claims in published maps and institutional affiliations.

## Figures and Tables

**Figure 1:**
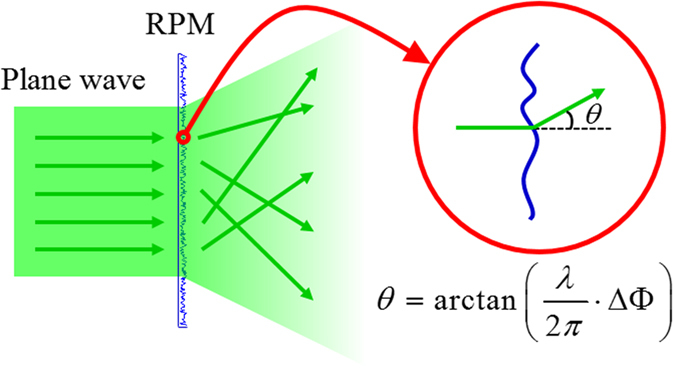
Schematic diagram of light scattering caused by the random phase mask.
